# Prognostic significance of HER3 and HER4 protein expression in colorectal adenocarcinomas

**DOI:** 10.1186/1471-2407-6-46

**Published:** 2006-02-28

**Authors:** Panteleimon Kountourakis, Kitty Pavlakis, Amanda Psyrri, Dimitra Rontogianni, Nikolaos Xiros, Efstratios Patsouris, Dimitrios Pectasides, Theofanis Economopoulos

**Affiliations:** 1 Second Department of Internal Medicine-Propaedeutic, Athens University Medical School, Attikon university Hospital, Athens, Greece; 2 Yale Cancer Center, New Haven, CT, USA; 3 Pathology Department, Athens University Medical School, Greece; 4 Pathology Department, Evangelismos Hospital, Athens-Hellas, Greece

## Abstract

**Background:**

Colorectal cancer remains a major cause of cancer mortality in the Western world. A limited number of studies  has been conducted in respect of Her-3 and Her-4 expression and their correlation with  clinical parameters and prognosis in colorectal carcinomas . In this study we sought to determine the pattern and the prognostic significance of HER-3 and HER-4 in colorectal adenocarcinoma.

**Methods:**

We studied HER-3 and HER-4 protein expression in106 paraffin embedded specimens of primary colorectal tumors using immunohistochemistry. The pattern and protein expression levels of HER-3 and HER-4 were correlated with several clinical and pathological parameters.

**Results:**

HER-3 staining displayed membranous and cytoplasmic expression pattern in 18 (17%) and 30 samples (28,3%), respectively. HER-4 membranous and cytoplasmic expression was found in 20 (18,9%) and 32 samples (30,2%), respectively. Specimens regarded as positive for HER-3 cytoplasmic expression were associated with moderate tumor grade (p = 0,032) and older median age (p = 0,010). Specimens regarded as positive for HER-4 membranous protein expression were associated with involved lymphnodes (p = 0,0003). Similar results were obtained when considering Her-3 and Her-4 protein expression irrespective of their cellular localization. There was no correlation between the expression of HER-3 and HER-4 and patients outcome.

**Conclusion:**

HER-4 membranous protein expression was found to predict for lymph nodes positivity in this cohort of patients with colorectal cancer.HER-4 expression status may identify tumors with aggressive biological behavior and increased metastatic potential.

## Background

Colorectal cancer remains a major cause of cancer mortality in the Western world both in men and women. It is the second most common malignancy (13.1%) and the second most common cause of cancer death in Europe[1]. The incidence of colorectal cancer has been increasing worldwide rapidly over the past decades.

The stage of the disease is the most important factor predicting the treatment's outcome[2]. At presentation, 30% of the patients have locally advanced cancer or metastatic disease, which discourages surgical care. Even among patients who undergo curative resection, approximately a 50% dies within five years. The role of adjuvant chemotherapy is clear in Dukes' C, but is controversial for Dukes' B cancers[3]. Although TNM (tumor-nodes-metastasis) classification is useful for staging patients and selecting them for specific treatment, it is not sufficient, as many patients at the same stage may have various outcomes. Therefore, there is a great need to identify useful prognostic markers to guide treatment decisions and/or to develop more effective treatments.

The transmembrane receptors that have action as tyrosine kinases play an important role in the pathogenesis of cancer in solid organs[4]. The type I receptor tyrosine kinase family comprises four homologous members: erbB1 (EGFR or HER-1), erbB2 (HER-2/neu), erbB3 (HER-3) and erbB4 (HER-4)[5].

The phosphorylation of these receptors through ligand binding and homo- or hetero-dimerization activates a cascade of signaling pathways that include: 1)the stress activated protein kinase pathway, involving protein kinase C and Jak/Stat, 2) the ras-raf-mitogen activated protein kinase pathway (MAP Kinase), 3) the protein serine/threonine kinase Akt pathway. These routs regulate cellular growth, differentiation, proliferation, angiogenesis and apoptosis[6].

Ten genes have been identified to encode HER-ligands. Out of these, the neuregulins(or heregulins) bind to HER-3 and HER-4 while betacellulin, epiregulin and heparin-binding EGF bind to HER-4[7].

HER-3 gene is located on chromosome 12q13 and encodes a 160 KDa transmembrane glycoprotein, that has no functional kinase domain and requires the dimerization with another receptor to activate downstream signal transduction pathways[8]. It's found to be overexpressed in various organs including breast, lung, pancreas and stomach. HER-4 gene is located on chromosome 2q33.3–34 and encodes a 180 KDa transmembrane glycoprotein[9]. HER-4 plays a crucial role in several important pathological processes – such as malignancy and heart disease – and in the development and differentiation of various tissues, especially cardiovascular, neural system and mammary glands[10].

There is limited data regarding the prognostic role of HER-3 and HER-4 in human cancer and these prove to be controversial. Some reports associate their overexpression with short while others with longer survival, thus, extended studies are required to ascertain definitively their prognostic value [11-13]. A limited number of studies has been conducted in respect of Her-3 and Her-4 expression and their correlation with clinical parameters and prognosis in colorectal carcinomas. Little therefore is known about their role in the pathogenesis of colorectal cancer. This study was conducted in order to investigate the immunohistochemical expression of Her-3 and Her-4 in colorectal carcinoma, to clarify their role in the prognosis of these carcinomas and to investigate the possible correlation with various clinical and pathological parameters.

## Methods

### Patients and tumor specimens

One hundred and six (106) formalin fixed and paraffin embedded colorectal cancer specimens, diagnosed at the Pathology Department of Evangelismos Hospital in Athens from 1997 to 2003, were studied. These specimens were collected from patients treated at the Oncology Unit of the Second Department of Internal Medicine – Propaedeutic, University of Athens. The clinical information of these patients derives from the medical records of the Department. Among the 106 patients, 103 had undergone surgical resection of the tumor; the remaining 3 patient's diagnosis was established by colonoscopy and consecutive biopsy. The evaluation of the 3 patients' preoperative imaging revealed metastasis. Since no surgery was performed, no information about the tumor size and the status of lymph nodes was available. None of the 106 patients had received chemotherapy or radiation therapy prior to surgery or died in the peri- or post-operative time period (within 45 days after surgery). The patients were staged using the Astler Coller – modified Dukes system. There were 30 patients (28.3%) with Dukes B, 51 patients (48.1%) with Dukes C and 25 patients (23.6%) with Dukes D. Dukes A patients were not included in the study since they received no further treatment. Postoperative chemotherapy with irinotecan, leucovorin and 5-fluorouracil (IFL) was administered to the whole group of patients. Although there is controversy concerning the role of adjuvant IFL chemotherapy in Dukes' B and Dukes' C cancers, the selected treatment was part of an adjuvant protocol or as first line treatment for metastatic disease of the Hellenic Cooperative Oncology Group (HECOG). Informed consent was obtained from all the patients and the protocol was approved by Attikon university hospital ethics commitee. All patients received regular follow-ups and survival data as of March 2005 were ascertained through the patients' records. The median duration of the follow-ups was 31 months (range 7–125 months).

### Immunohistochemistry

All the available hematoxylin and eosin stained slides of surgical specimens were reviewed and representative paraffin blocks for each case were selected for immunohistochemical study. Two serial sections of 4 μm thick were cut from each block and placed onto Super Frost Plus glass-slides. Following deparaffinization in xylene, the slides were rehydrated and washed in Tris Buffered Saline (TBS). The endogenous peroxidase activity was quenched by 10 min incubation in a mixture of 3% hydrogen peroxide solution in 100% methanol (Sigma). Slides were cleared with TBS and placed at room temperature for 1 hour. They were then incubated with monoclonal mouse antibody to human Her-3 protein (MAb-MsS-725-P, Neomarkers, Fremont, CA) at 1/35 dilution and with monoclonal mouse antibody to human Her-4 protein (Ab-4, HFR-1, Neomarkers, Fremont, CA) at 1/100 dilution overnight at +4°C. Slides were washed three times in TBS. The signal was visualized using the DAKO-EnVision Kit (k 5007 HRP Rabbit/Mouse DAB+) for 60 min. After washing with TBS, slides were kept in diaminobenzidine tetrahydrochloride for 7 min then counterstained with Mayers hematoxylin. Sections of breast carcinoma and striated muscle were used as positive controls. Slides were treated as negative controls by omitting the primary antibody.

### Scoring system

Sections were examined using light microscopy by two independent observers (K.P. and D.R) who were unaware of the clinicopathological data. Interobserver variation was resolved by simultaneous dual re-evaluation. Both membranous and cytoplasmic immunostaining was evaluated semiquantitatively.

**Figure 1:**
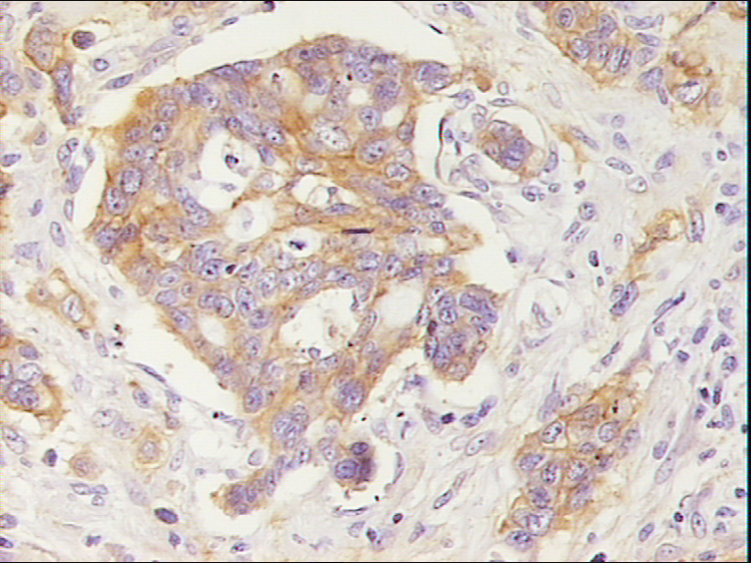
HER-3 strong membranous and weak to moderate cytoplasmic staining (× 200).

**Figure 2:**
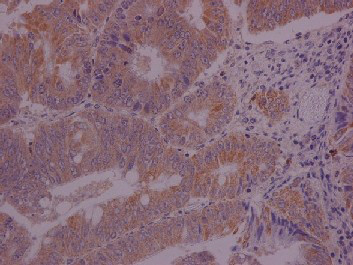
Positive diffused and granular exclusively cytoplasmic staining for HER-4 (x400)

Membranous staining was considered positive (+) when more than 1% of tumor cells were stained, irrespective of whether the staining pattern was complete circumferential or not (Fig. 1). When less than 1% of tumor cells were stained it was considered negative (-).Cytoplasmic staining was scored as 0, no staining or weak staining in <10% of tumor cells; 1+, weak immunostaining in >10% of tumor cells; 2+, moderate immunostaining in >10% of tumor cells and 3+, strong immunostaining in >10% of tumor cells. Staining pattern was either granular or diffuse (Fig. 2). Scores of 0 and 1+ indicate a negative tumor, while scores of 2+ and 3+ were regarded as positive.

**Figure 3:**
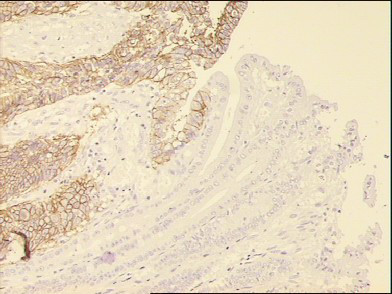
Complete circumferential membranous staining of HER-3 as opposed to the absence of immunoreaction in the adenomatous part of lesion (× 200)

Tumors were considered positive for both, membranous and cytoplasmic staining by using the above mentioned criteria, irrespective of whether the expression of the antibodies was localized in the same cell or in different groups of tumor cells (Fig. 3).

### Statistical analysis

Categorical characteristics were summarized as percentages and their association with HER-3 and HER-4 overexpression was assessed using the Pearson χ^2 ^test or the Fisher exact test[14]. Continuous characteristics comparisons were performed using the Mann- Whitney test. The probability of survival was calculated by the Kaplan- Meier method and differences in survival were assessed by the log- rank test[15]. All statistical tests were performed on a = 0.05 level of significance. Analysis was performed using SPSS 11.0.1 for windows.

## Results

One hundred and six (106) colon carcinomas samples were examined. Sixty six patients were males (62.3%) and forty patients were females (37.7%). The median age of the patients was 63 years (35- 79 years). Patients' median follow up time was 31 months (7- 125 months). In this period of time, 33 deaths and 38 relapses were observed. The 3-year survival rate was 72.4%, and the 5-year survival rate was 45.7%.

Eighteen samples (17%) out of one hundred and six were evaluated as positive for HER-3 membranous protein expression. Cytoplasmic staining for HER-3 was evaluated as follows: 39 specimens (36,8%) were negative for HER-3 expression,37 (34,9 %) were scored as 1+, 20 (18,9%) were scored as 2+, while 10 (9,4%)were scored as 3+. Scores of 2+ and 3+ were regarded as positive expression.

Twenty samples (18,9%) were positive for HER-4 membranous protein expression. Cytoplasmic staining for HER-4 was evaluated as follows:32 samples (30,2%) were negative for HER-4 expression,42 (39,6%) were scored as 1+, 29 (27,4%) were scored as 2+, while 3 (2,8%) were scored as 3+. Scores of 2+ and 3+ were regarded as positive expression.

When considering Her-3 and Her-4 protein expression as positive irrespective of the cellular localization, 37 specimens (34.9%) were found to be positive for Her-3 and 40 specimens (37.7%) positive for Her-4.

Statistical analysis revealed no relationship between membranous Her-3 protein expression and any of the clinicopathological parameters under evaluation (Table 1). Patients regarded as negative for HER-3 cytoplasmic expression had smaller median age than those with positive HER-3 cytoplasmic expression (61 versus 66 years, p = 0,010). All the patients regarded as positive for HER-3 cytoplasmic expression, had moderately differentiated tumors (p = 0,032) (Table 1).

The majority of positive samples for HER-4 membranous protein expression (95%) were associated with involved lymph nodes (p = 0,0003). Patients regarded as negative for HER-4 membranous expression were associated with Dukes' stage B (p = 0.010) (Table 2). There was no association between HER-4 cytoplasmic protein expression and any clinicopathological parameter (Table 2).

For tumors that were considered as positive irrespective of the cellular localization of each marker, statistical analysis revealed that Her-3 expression was associated with an older median age group (p = 0.009) and with moderately and poorly differentiated tumors (p = 0.008 and p = 0.048 respectively). Her-4 positivity was related with involved lymphnodes (p = 0.010) (Table 3). There was no association with patients' survival as analysed by Kaplan Meier's curves (data non shown).

**Figure 4:**
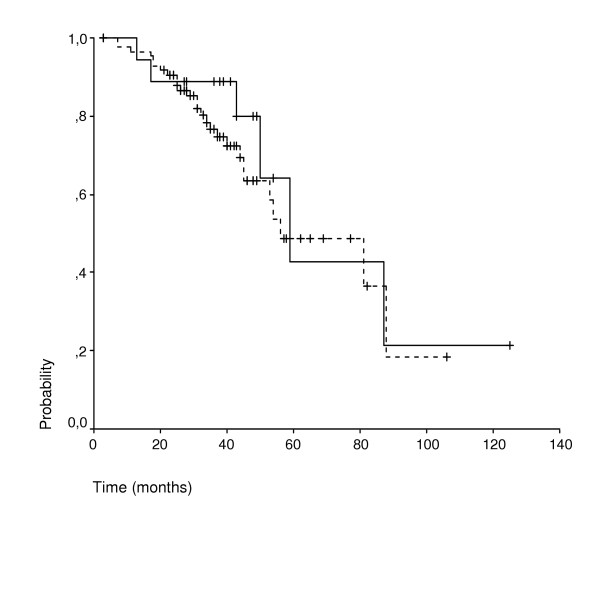
Kaplan Meier curves for the overall survival of patients with positive (continuous line) and negative (dotted line) HER-3 membranous expression.

**Figure 5:**
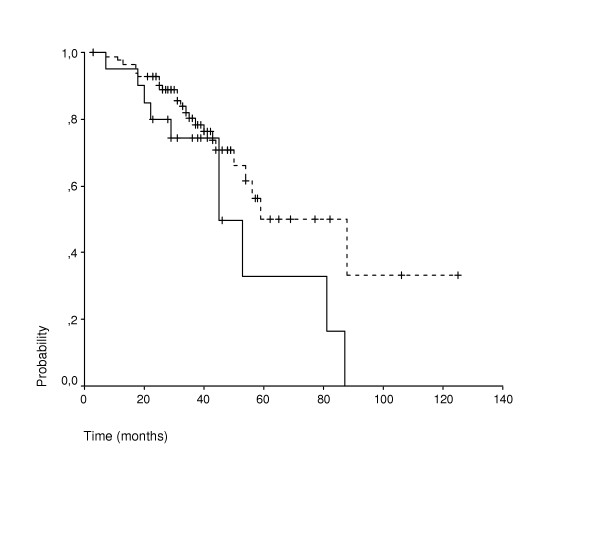
Kaplan Meier curves for the overall survival of patients with positive (continuous line) and negative (dotted line) HER-4 membranous expression.

**Figure 6:**
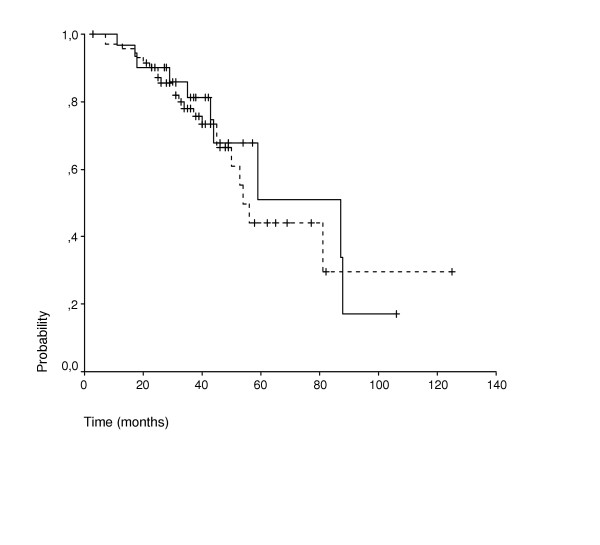
Kaplan Meier curves for the overall survival of patients with positive (continuous line) and negative (dotted line) HER-3 cytoplasmic expression.

**Figure 7:**
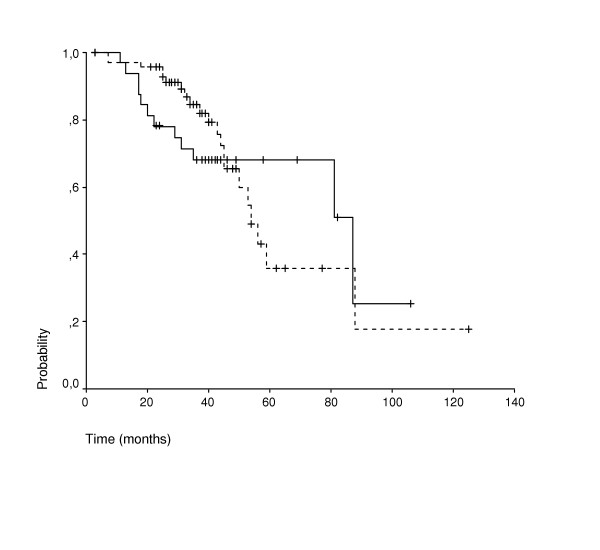
Kaplan Meier curves for the overall survival of patients with positive (continuous line) and negative (dotted line) HER-4 cytoplasmic expression.

Membranous or cytoplasmic expression of HER-3 and HER-4 was not associated with patients' survival as analysed by Kaplan Meier's curves (Figures 4, 5, 6, 7). Coexpression for HER -3 and HER-4 was observed in 3 out of 106 cases (2,8%) with membranous staining (k = 0.025, p = 0,793) and in 15 out of 106 cases (14,2%) with cytoplasmic staining (k = 0.271, p = 0,005). There was no statistically significant difference in patients characteristics or overall survival with respect to Her-3 and Her-4 coexpression as compared to the expression of each antibody alone.

## Discussion

Colorectal malignancies remain one of the major causes of cancer death. The last decade newer cytotoxic agents such as oxaliplatin and irinotecan have shown promise in the adjuvant setting. The administration of these agents has increased the overall survival rate as well as the time to progression in patients with metastatic cancer[16,17]. Despite significant improvements in traditional chemotherapy regimens, over the last years, the main efforts of research have focused on the use of targeted therapy.

The low molecular weight tyrosine kinase inhibitors and the monoclonal antibodies against EGFR are in clinical development[18,19]. These strategies have shown promise; more efforts focus on the identification of new molecular prognostic markers and the development of molecular targeted therapies against colorectal cancer.

The type I tyrosine kinase receptors and the associated signal transduction pathways have a crucial role in cancer biology and are attractive targets for cancer therapy. Out of the four members of the type I receptor tyrosine kinase family, EGFR and Her-2 have been widely studied on several human tumors, mostly breast and colon carcinoma. In most studies their protein expression as evaluated by immunohistochemical techniques was found to be related to poor prognostic factors[20,21]. On the contrary the role of Her-3 and Her-4 in the pathobiology of carcinomas has not yet been clarified. Most of the studies were conducted on breast carcinomas and the results have proven to be controversial. Most investigators revealed that HER-3 protein expression on breast carcinomas was associated with poor prognostic factors such as large tumor size, high grade of malignancy and lymph node metastasis[22,23]. However, others have found that HER-3 expression was associated with an improved 10- year survival as well as estrogen receptor (ER) positivity, which is a favorable prognostic factor for breast cancer[24]. HER-4 protein overexpression on breast carcinomas was associated in several studies with the longer survival of patients, ER positivity, low proliferation rate and low probability of recurrence, while other studies have correlated HER-4 overexpression with negative prognostic factors [25-27]. Results in human breast cancer cell lines are also conflicting (28,29).

In colorectal cancer, only a few studies have investigated the protein expression of Her-3 and Her-4. From the obtained data, the incidence of HER-3 expression in colorectal cancer ranges from 36 % to 89 % (30, 31). Poller et al. (32) revealed a 88,8% positive rate for HER-3 expression, both mebranous and cytoplasmic. Rajkumar et al. (33) detected a 68,75 % rate for HER-3 expression, predominantly cytoplasmic. Maurer and co-workers (31) demonstrated that HER-3 is expressed in 89 % in primary colorectal carcinomas and the immunoreactivity was both membranous and cytoplasmic. Kapitanovic et al.(34) revealed a 78 % rate for HER-3 expression, which was exclusively cytoplasmic. Lee et al.(30) detected a 36 % rate for membranous HER-3 expression and a 22 % rate for membranous HER-4 expression. To the best of our knowledge this was the only study conducted on Her-4 protein expression in colon carcinoma

In our study, both membranous and cytoplasmic Her-3 and Her-4 protein expression was identified. The rates of expression for Her-3 and Her-4 respectively were as follows: 17% membranous, 28.3 % cytoplasmic and 18.9% membranous, 30.2% cytoplasmic. Both membranous and cytoplasmic staining was found in 11.3% of Her-3 and 14.1% of Her-4 positive cases. As opposed to the five studies already mentioned, we evaluated protein expression not only irrespective of the cellular localization of the antibody but also on different cellular compartment (membrane or cytoplasm) separately, assuming that this phenomenon might be related to divergent functional properties of the genes.

In their study, Maurer et al.(31) suggested a growth- enhancing role of Her-3 in colorectal cancer. They speculated that extreme Her-3 immunostaining was associated with tumors presenting mucus production, which is a sign of advanced tumor disease. Overexpression of Her-3 was also found in the invading edge of the tumor as well as in the transition zone between adenomatous and cancerous tissue (Fig. 3). These findings were considered to indicate the role of Her-3 in tumor progression. Moreover, Kapitanovic et al.(34) revealed that patients regarded as negative for HER-3 expression survived significantly longer than patients with HER-3 expression (p < 0.05). On the other hand, the results of Lee et al.(30) demonstrated that HER-3 overexpression was correlated with patients in the early stages of the disease (p < 0.0001). In our study, Her-3 membranous expression was not related to any of the clinicopathological parameters under evaluation while positive cytoplasmic Her-3 protein expression was found mostly in moderately differentiated tumors(p = 0.032) and in the older age group (p = 0.010). When cases with both membranous and cytoplasmic staining were considered as positive, then also poorly differentiated tumors (p = 0.048) were found to have a statistical significant overexpression of Her-3 protein. Due to the small number of cases (3 highly differentiated – 8 poorly differentiated) these findings are not sufficient to support any possible role of Her-3 in tumor progression.

Regarding Her-4 protein expression on colorectal cancer, only Lee et al have recently conducted a detailed study. In their series, 22% of the cases showed membranous overexpression of Her-4. This incidence tended to be higher in later stage than in early stage cancers. This is in accordance with our observations. In our study, the majority of positive samples for HER-4 membranous protein expression (95%) were associated with involved lymph nodes (p = 0,0003). The same results were obtained when cases with both membranous and cytoplasmic expression of Her-4 were considered as positive (p = 0.010). Patients who tested negative for HER-4 membranous expression were associated with Dukes' stage B cancer (p = 0.010).

The coexpression of both Her-3 and Her-4 membranous and cytoplasmic immunostaining for each marker separately was not found to be related to any clinicopathological parameter.

## Conclusion

Our findings do not support any major role between Her-3 protein expression and tumor biology in colon carcinoma. Judging from the two existing studies on Her-4, the study of Lee et. al (30) and our own, there is strong evidence that Her-4 membranous protein expression in colon carcinoma, might indicate at tumors with more aggressive biological behavior. If this proves to be right, then some patients could be favored from the administration of pan-Her inhibitors. Certainly, our relatively small sample may not have been enough to detect differences. Larger studies with extended follow-ups will be required to ascertain the prognostic value of Her-3 and Her-4 expression in colorectal carcinomas. Moreover, in this study, only HER-3, HER-4 part of the signaling system was evaluated. Estimating the expression of all four members of the Her family as well as of an array of receptor-specific ligands would probably give more information on some steps of the signal transduction pathway and on the clinical significance of the interactions between the different receptors.

## Competing interests

The author(s) declare that they have no competing interests.

## Authors' contributions

P.K. participated in study design, collected specimens and clinical data, interpreted the data, carried out functional studies, drafted the manuscript.

K.P. participated in study design, carried out the functional studies, evaluated immunohistochemistry, interpreted the data

A.P. participated in manuscript preparation.

D.R. provided the specimens, evaluated immunohistochemistry.

N.X. provided clinical data.

E.P. revised the manuscript.

D.P. supervised and controlled the whole study.

T.E. supervised and controlled the whole study.

All Authors read and approved the final manuscript.

## Pre-publication history

The pre-publication history for this paper can be accessed here:



## Figures and Tables

**Table 1:** Patient clinicopathological data and HER-3 membranous and cytoplasmic expression

	HER-3 membranous expression			HER-3 cytoplasmic expression		
**Characteristic**	(+) (n= 18)	(-) (n= 88)	p	(+) (n= 30)	(-) (n= 76)	P

**Age Median (range)**	68 (53 – 75)	63 (35 – 79)	0.069	66 (52 – 76)	61 (35 – 79)	0.010
**Sex**						
Male	10 (55.6%)	56(63.6%)	0.519	15 (50.0%)	51 (67.1%)	0.112
Female	8 (44.4%)	32(36.4%)		15 (50.0%)	25 (32.9%)	
Size	5 (2 – 12)	5 (1.5 – 12)	0.959	5 (2 – 10)	5 (1.5 – 12)	0.525
**Lymph nodes status**						
(+)	13 (72.2%)	56(65.9%)	0.603	20 (66.7%)	49 (67.1%)	0.964
(-)	5 (27.8%)	29(34.1%)		10 (33.3%)	24 (32.9%)	
**Grade**						
Well	-	3 (3.4%)	0.999	-	3 (3.9%)	0.556
Moderate	18 (100%)	77(87.5%)	0.205	30 (100.0%)	65 (85.5%)	0.032
Poor	-	8 (18%)	0.346	-	8 (10.5%)	0.101
**Dukes' stage**						
B	4 (22.2%)	26(29.5%)	0.530	9 (30.0%)	21 (27.6%)	0.807
C	11 (61.1%)	40(45.5%)	0.226	14 (46.7%)	37 (48.7%)	0.851
D	3 (16.7%)	22(25.0%)	0.448	7 (23.3%)	18 (23.7%)	0.969
**Site**						
Proximal colon	4 (22.2%)	25(28.4%)	0.774	7 (23.3%)	22 (28.9%)	0.534
Distal colon	9 (50.0%)	28(31.8%)	0.140	10 (33.3%)	27 (35.5%)	0.796
Rectum	5 (27.8%)	35(39.8%)	0.339	13 (43.3%)	27 (35.5%)	0.406

**Table 2:** Patient clinicopathological data and HER-4 membranous and cytoplasmic expression

	HER-4 membranous expression			HER-4 cytoplasmic expression		
**Characteristic**	(+) (n= 20)	(-) (n= 86)	p	(+) (n = 32)	(-) (n= 74)	P

**Age Median (range)**	64 (40 – 75)	63 (35 – 79)	0.906	64 (40 – 76)	63 (35 – 79)	0.656
**Sex**						
Male	13 (65.0%)	53 (61.6%)	0.779	20 (62.5%)	46 (62.2%)	0.974
Female	7 (35.0%)	33 (38.4%)		12 (37.5%)	28 (37.8%)	
Size	5 (1.6 – 8.5)	5 (1.5 – 12)	0.383	5 (2 – 10)	5 (1.5 – 12)	0.128
**Lymph nodes status**						
(+)	19 (26.8%)	50 (60.2%)	0.003	25 (78.1%)	44 (62.0%)	0.107
(-)	1 (5.0%)	33 (39.8%)		7 (21.9%)	27 (38.0%)	
**Grade**						
Well	-	3 (3.5%)	0.999	-	3 (4.1%)	0.552
Moderate	17 (85.0%)	78 (90.7%)	0.431	31 (96.9%)	64 (86.5%)	0.167
Poor	3 (15.0%)	5 (5.8%)	0.171	1 (3.1%)	7 (9.5%)	0.430
**Dukes' stage**						
B	1 (5.0%)	29 (33.7%)	0.010	7 (21.9%)	23 (31.1%)	0.334
C	11 (55.0%)	40 (46.5%)	0.494	14 (43.8%)	37 (50.0%)	0.554
D	8 (40.0%)	17 (19.8%)	0.078	11 (44.4%)	14 (18.9%)	0.085
**Site**						
Proximal colon	4 (20.0%)	25 (29.1%)	0.412	11 (34.4%)	18 (24.3%)	0.287
Distal colon	9 (45.0%)	28 (75.7%)	0.293	11 (34.4%)	26 (35.1%)	0.940
Rectum	7 (61.6%)	33 (38.4%)	0.779	10 (31.2%)	30 (40.5%)	0.365

**Table 3:** Patient clinicopathological data and HER-3, HER-4 expression

	HER- 3 expression (membranous or cytoplasmic)			HER- 4 expression (membranous or cytoplasmic)		
**Characteristic**	(+) (n= 37)	(-) (n= 69)	p	(+) (n= 40)	(-) (n= 66)	p

**Age Median (range)**	65 (52 – 76)	61 (35 – 79)	0.009	64.5 (40 – 76)	61 (35 – 79)	0.189
**Sex**						
Male	19 (51.4%)	47 (68.1%)	0.098	24 (60%)	42 (63.6%)	0.836
Female	18 (48.6%)	22 (31.9%)		16 (40%)	24 (36.4%)	
Size	5 (2 – 12)	5 (1.5 – 12)	0.425	5 (2 – 12)	5 (1.5 – 12)	0.195
**Lymph nodes status**						
(+)	25 (67.6%)	44 (63.8%)	0.999	33 (82.5%)	36 (54.5%)	0.010
(-)	12 (32.4%)	22 (31.9%)		7 (17.5%)	27 (40.9%)	
**Grade**						
Well	-	3 (4.2%)	0.550	-	3 (4.5%)	0.288
Moderate	37 (100%)	58 (84.1%)	0.008	37 (92.5%)	58 (87.9%)	0.529
Poor	-	8 (11.6%)	0.048	3 (7.5%)	5 (7.6%)	0.999
**Dukes' stage**						
B	11 (29.7%)	19 (27.5%)	0.824	7 (17.5%)	23 (34.8%)	0.075
C	18 (48.6%)	33 (47.8%)	0.999	20 (50%)	31 (47%)	0.842
D	8 (21.6%)	17 (24.6%)	0.813	13 (32.5%)	12 (18.2%)	0.104
**Site**						
Proximal colon	10 (27%)	19 (27.5%)	0.999	12 (30%)	17 (25.8%)	0.658
Distal colon	13 (35.1%)	24 (34.8%)	0.999	14 (35%)	23 (34.8%)	0.999
Rectum	14 (37.8%)	26 (37.7%)	0.999	26 (39.4%)	14 (35%)	0.684

Proximal colon:cecum, ascending, transverse colon. Distal colon:descending, sigmoid colon
